# Withdrawal Note: Microbiota dysbiosis influences immune system and muscle pathophysiology of dystrophin-deficient mice

**DOI:** 10.1038/s44321-026-00446-0

**Published:** 2026-06-03

**Authors:** Andrea Farini, Luana Tripodi, Chiara Villa, Francesco Strati, Amanda Facoetti, Guido Baselli, Jacopo Troisi, Annamaria Landolfi, Caterina Lonati, Davide Molinaro, Michelle Wintzinger, Stefano Gatti, Barbara Cassani, Flavio Caprioli, Federica Facciotti, Mattia Quattrocelli, Yvan Torrente

**Affiliations:** 1 Neurology Unit, Fondazione IRCCS Ca’ Granda Ospedale Maggiore Policlinico, Milan, Italy; 2 Stem Cell Laboratory, Department of Pathophysiology and Transplantation, Dino Ferrari Center, University of Milan, Milan, Italy; 3 Mucosal Immunology Lab, Department of Experimental Oncology, IEO-European Institute of Oncology, Milan, Italy; 4 Humanitas University, Milan, Italy; 5 Humanitas Clinical and Research Center IRCCS, Milan, Italy; 6 Translational Medicine – Department of Transfusion Medicine and Hematology, Fondazione IRCCS Ca’ Granda Ospedale Maggiore Policlinico, Milan, Italy; 7 Department of Medicine, Surgery and Dentistry, Scuola Medica Salernitana, University of Salerno, Baronissi, Italy; 8 Theoreo Srl, Spinoff Company of the University of Salerno, Montecorvino Pugliano, Italy; 9 Center for Surgical Research, Fondazione IRCCS Ca’ Granda, Ospedale Maggiore Policlinico, Milan, Italy; 10 Molecular Cardiovascular Biology Division, Heart Institute, Cincinnati Children’s Hospital Medical Center, Cincinnati, OH USA; 11 Department of Pediatrics, University of Cincinnati College of Medicine, Cincinnati, OH USA; 12 Department of Medical Biotechnologies and Translational Medicine, Universita Degli Studi di Milano, Milan, Italy; 13 Unit of Gastroenterology and Endoscopy, Department of Pathophysiology and Transplantation, Università degli Studi di Milano, Fondazione IRCCS Ca’ Granda, Ospedale Policlinico di Milano, Milan, Italy; 14 Present Address: SciLifeLab, Department of Microbiology, Tumor and Cell Biology, Karolinska Institutet, Solna, Sweden

## Abstract

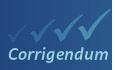

**Author withdrawal to**: *EMBO Molecular Medicine* (2023) 15:e16244. 10.15252/emmm.202216244 | Published online 19 December 2022

The journal was made aware of citation errors in the manuscript. The journal conducted an internal assessment and performed an image screening of the published figures. This assessment identified possible image reuse. The authors were contacted for clarification and provided the corresponding original source data for the affected figures, along with early manuscript drafts containing intact metadata. These materials demonstrate that the citation errors were not present in earlier iterations of the manuscript and were introduced during the manuscript preparation process prior to submission.

Given the extent of the textual revisions required to address the citation issues, a corrected version of the article, titled *“Microbiota Dysbiosis Influences Immune System and Muscle Pathophysiology of Dystrophin Deficient Mice,”* has been accepted for publication in *EMBO Molecular Medicine* (Farini et al, 2026). This withdrawal is issued to avoid confusion between the originally published version and the corrected version of the manuscript.

**The manuscript is withdrawn and replaced. Affected citations as well as Figures 6E and EV2 are corrected**.

**Corresponding source data are published with this withdrawal statement.**

## Author statement

Following notification from the journal, we undertook a detailed reconstruction of the chronology of all Word files associated with the manuscript to determine the origin of the citation discrepancies.

Our assessment identified the root cause as an interoperability failure between different versions of EndNote used during manuscript preparation. The initial version of the manuscript was created on a personal computer using EndNote 20. This file was later opened and edited on a departmental computer running the much older EndNote X7. The version mismatch resulted in corruption of the bibliography, including random or incorrect reference insertions.

Regarding the figure issues, we acknowledge that incorrect images were inadvertently included for Figure EV2 (Western blot) and Figure 6E (C57Bl 3 m and MDX 3 m). We sincerely apologise for this oversight and have supplied the correct files.

To ensure full transparency and uphold scientific integrity, we have provided all raw data corresponding to these figures, together with detailed metadata indicating when each image was originally generated.

All authors agree to the withdraw and replacement of this article and apologise for any inconvenience caused.

## Supplementary information

Source data Fig. 6E

Source data Fig. EV2
